# Improving sensitivity to eye gaze cues in adolescents on the autism spectrum using serious game technology: A randomized controlled trial

**DOI:** 10.1002/jcv2.12041

**Published:** 2021-10-04

**Authors:** Jason W. Griffin, Charles F. Geier, Joshua M. Smyth, K. Suzanne Scherf

**Affiliations:** ^1^ Department of Psychology Pennsylvania State University University Park Pennsylvania USA; ^2^ Department of Human Development and Family Studies Pennsylvania State University University Park Pennsylvania USA; ^3^ Departments of Biobehavioral Health and Medicine Pennsylvania State University University Park Pennsylvania USA

**Keywords:** autism spectrum disorders, eye gaze, social cognition, intervention

## Abstract

**Background:**

Perceiving and interpreting eye gaze cues is foundational for social cognition and social interactions because it involves the ability to use eye gaze direction to predict the actions and intentions of others. Autism is a disability that impacts social interactions. A diagnostic symptom of autism is difficulty understanding eye gaze cues as social signals. This deficit has long‐term consequences for understanding goal‐directed behavior, language learning, and social communication. We hypothesize that targeted intervention methods designed to improve sensitivity to eye gaze cues may begin to treat core symptoms of autism and potentially alter multiple aspects of social functioning. Social Games for Autistic Adolescents (SAGA) is a serious computer game intervention designed to improve sensitivity to eye gaze cues. Serious games improve targeted skills with the goal of enhancing real‐life outcomes. In SAGA, participants progress through a narrative storyline and interact with animated characters. In so doing, they implicitly discover that eye gaze cues are useful for guiding their own goal‐directed behavior to solve problems in the game.

**Methods:**

We evaluated the feasibility and effectiveness of SAGA in a hybrid phase 1/2, randomized controlled trial. Forty adolescents on the autism spectrum were randomized to either the treatment or standard care control group. Adolescents in the treatment group were asked to play SAGA for 30‐min sessions at home 3 times a week over 10 weeks.

**Results:**

A group × time interaction revealed that the treatment group developed increasing sensitivity to human eye gaze cues, whereas the standard care group did not. Participants who experienced a sufficient dose of gameplay showed larger treatment‐related improvements. Critically, increases in sensitivity to human eye gaze cues were associated with improvements in social skills.

**Conclusions:**

This accessible, scalable, and affordable intervention shows promise as an effective tool for improving the ability to interpret and understand eye gaze cues and social skills in adolescents on the autism spectrum.

## Graphical abstract

Deficits in eye gaze processing impact social cognition and social interactions in autism, even for individuals in adolescence and adulthood. We designed a computerized, home‐based, serious game intervention to improve eye gaze processing in adolescents on the autism spectrum. Following 10 weeks of training, adolescents in the treatment group improved their ability to detect and understand human eye gaze cues.

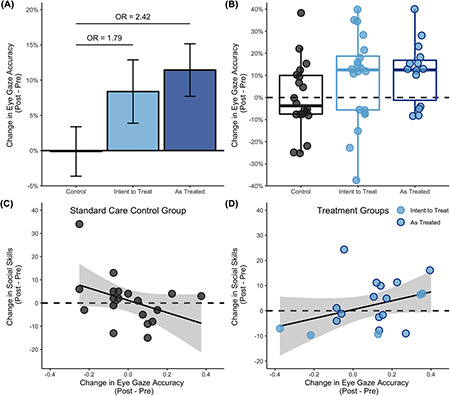

## INTRODUCTION

Autism spectrum disorder (ASD) is a neurodevelopmental disorder characterized by impairments in social communicative behavior (American Psychiatric Association, 2013). Core symptoms that reflect these impairments are deficits in social looking behaviors. In particular, people on the autism spectrum (Botha et al., 2021; Bury et al., 2020) have difficulty understanding shifts in eye gaze as social signals (i.e., eye gaze cues; see Guillon et al., 2014). This is problematic because perceiving and interpreting eye gaze cues is foundational for social cognition and social interactions, including deception, empathy, and theory of mind (for review see Emery, 2000).

**Key points**

- Deficits in eye gaze processing impact social cognition and social interactions in autism, even for individuals in adolescence and adulthood
- We designed a computerized, home‐based, serious game intervention to improve eye gaze processing in adolescents on the autism spectrum
- We tested the feasibility and effectiveness of this intervention in a randomized controlled trial with treatment and standard care groups
- Following 10 weeks of training, adolescents in the treatment group improved their ability to detect and understand human eye gaze cues, particularly for those who acquired a sufficient does of training, which was related to improvements in their social skills
- This intervention has the potential to compliment standard treatments for autism to improve social cognition and social interactions in multiplicative ways

At the most foundational level, shifts in gaze are used to communicate the relative importance of objects and people in the world. Interpreting these signals requires the abilities to follow the gaze shift and to understand the referential nature of visual behavior. This requires that one establish a psychological connection between the looker and the content (Brooks & Meltzoff, 2005) to understand that visual behavior is directed toward objects/content and involves the mental experience of seeing (Moore, 1999). Among typically developing individuals, sensitivity to shifts of gaze begins within days of birth (Farroni et al., 2004) and continues to develop through childhood (Brooks & Meltzoff, 2005) and adolescence (Riby & Hancock, 2009; Riby et al., 2013). Critically, sensitivity to eye gaze cues is foundational for the development of social communicative skills like joint attention (Moore & Dunham, 1995), but also more complex social skills like language (Tomasello & Farrar, 1986), and theory of mind (Frith & Frith, 1999).

Deficits in processing eye gaze cues are life‐long for people on the autism spectrum. They are present in infants later diagnosed with autism (Bedford et al., 2012) and persist through childhood (Falck‐Ytter et al., 2012), adolescence (Pickard & Ingersoll, 2015), and adulthood (Fletcher‐Watson et al., 2009). Both behavioral (Griffin & Scherf, 2020; Riby et al., 2013; Vivanti et al., 2011) and neuroimaging (Pelphrey et al., 2005; Sato et al., 2017) findings indicate that the deficits appear to be disproportionately related to understanding the referential nature of gaze, and/or assigning social relevance to gazed‐at objects. These deficits have consequences for understanding goal‐directed behavior (Guillon et al., 2014; Riby et al., 2013; Vivanti et al., 2011), as well as language learning and social communication in ASD (Gulsrud et al., 2014; Kasari et al., 2012). Therefore, this core phenotype of the ASD diagnosis disadvantages autistic individuals in a wide variety of social contexts (e.g., educational, social relationships, employment). They are missing critical nonverbal information that contextualizes and directs social interactions in these contexts that are critical to navigate for establishing functional independence. Helping them to perceive and interpret this information (whether they use it to direct social interactions themselves) may provide essential information so that they can determine how they want to act on it. As a result, we suggest that enhancing sensitivity to eye gaze cues is an important potential target for intervention in ASD that could improve social communicative behaviors, and ultimately social interactions, in multiplicative ways.

There is some indirect evidence to support this notion. Although there are no interventions designed to improve sensitivity to eye gaze cues specifically, many interventions have targeted improvements in joint attention behaviors, which include coordinated attention to the same object, object showing, and pointing between two people. A meta‐analysis of the joint attention interventions designed for young children on the autism spectrum indicates that there are measurable effects on social communication behaviors following these interventions (see Murza et al., 2016). This is promising evidence that leads us to hypothesize that targeting improvements in sensitivity to eye gaze cues specifically may improve a wider range of social communicative behaviors in ASD because of the foundational role that shifts in gaze play in social cognition and human social interactions more broadly.

To test this hypothesis, we designed and tested a “serious game” computer intervention in a randomized controlled trial (RCT) for adolescents on the autism spectrum. Serious games are intervention tools designed to improve targeted skills (including those that are difficult and not intrinsically rewarding for participants), with the goal of enhancing real life outcomes (Whyte et al., 2015). They integrate educational objectives with evidence‐based game mechanics known to support learning and generalization of learning (Whyte et al., 2015). The Social Games for Adolescents with Autism (SAGA) intervention is designed to scaffold learning about the referential understanding of eye gaze cues in simulated social interactions with computer‐animated human characters, which are embedded in an age‐appropriate narrative storyline (Scherf et al., 2018). Players must discover that eye gaze cues can be used to guide their own goal‐directed behavior to solve problems in the game. This simulates the way eye gaze cues are discovered developmentally and used in the real world.

It is important to note that the goal of this intervention is to support implicit learning about how eye gaze cues are used as nonverbal social cues to guide and contextualize social interactions. It is very similar to the goal of helping individuals on the spectrum learn to perceive and interpret facial expressions (e.g., Golan et al., 2010). Such learning can potentially provide individuals on the spectrum with important information about the actions and intentions of others, which can facilitate their own decisions about how to act in the context of social interactions. Importantly, the intervention does not force participants to employ a particular social communicative behavior (e.g., look at faces or eyes). It only provides opportunities for learning in a safe environment to learn new skills. Finally, given the complexity of ASD as a disability, we acknowledge that a multidimensional approach to intervention will likely be most successful. Therefore, we envision that many other types of intervention approaches, including parent‐directed and public information‐oriented interventions, could be highly compatible with our approach.

We designed and tested the intervention with adolescents on the autism spectrum for several reasons. First, adolescence is a potentially vulnerable period of development in ASD when developmental trajectories decline or plateau, especially regarding the processing of social information, making it an important time for intervention (for review see Picci & Scherf, 2014). Second, social looking behavior may change in important ways during adolescence as individuals transition into new social roles with their peers in ways that require a new attentional focus on faces and eye gaze. Third, adolescents are old enough to tolerate a protocol that involved several hours of training each week over the course of 10 weeks, which is the minimal amount of training we hypothesized would be required to observe emerging changes in sensitivity to eye gaze cues.

The objective of this study was to evaluate the feasibility and effectiveness of SAGA for improving sensitivity to eye gaze cues and social skills in adolescents on the autism spectrum. The study was an experimental RCT in which participants were randomized to the serious game treatment group or a standard care control group. We predicted that scaffolded social interactions with game avatars would increase attention to shifts of gaze and improve understanding that gaze is referential in nature, which would generalize to support learning about human eye gaze in the real world in the treatment group. Operationally, this learning would be indicated by an increase in task performance (i.e., accuracy) on a gaze perception task following the intervention, which would not be observed in the standard care control group. Furthermore, participants in the treatment group who engaged in a minimum of 10 h of eye gaze tasks during training would show the largest improvements in the gaze perception task. We also predicted that improvements in sensitivity to eye gaze cues would be related to improvements in social skills and/or autism symptoms in the treatment, but not in the control group.

## METHODS

The protocol for this RCT has been published in its entirety (Scherf et al., 2018) and is included in the Supporting Information. It is also registered through clinicaltrials.gov (NCT02968225). The following description of the experimental methods is a brief overview of this extensive protocol.

### Trial design

SAGA was an experimental, two‐arm, phase 1/2 RCT. Adolescents on the autism spectrum were randomized to receive the serious game training or to continue standard care. This study was approved by the Institutional Review Board at Pennsylvania State University. An independent board monitored safety and examined interim feasibility and effectiveness results. The study protocol is published elsewhere (Scherf et al., 2018), and baseline data were published to compare performance of adolescents on the autism spectrum with that of age‐, sex‐, and IQ‐matched typically developing participants (Griffin & Scherf, 2020). No findings regarding the effectiveness of SAGA have been published.

### Participants and trial procedures

#### Recruitment

Adolescent participants, ages 10–18 years, and their families were recruited from two research databases: Interactive Autism Network Research database at the Kennedy Krieger Institute, Baltimore, and autismMatch at the Center for Autism Research, Philadelphia. Initial eligibility was assessed online or by phone (Scherf et al., 2018). Full eligibility was assessed in the Laboratory of Developmental Neuroscience at Pennsylvania State University (PSU). Participants were enrolled between December 2017 and February 2018 and followed up between March 2018 and May 2018.

#### Inclusion/exclusion criteria (Scherf et al., 2018)

Participants had to (1) have a diagnosis of ASD confirmed by the ADOS‐2 (Lord et al., 2012); (2) be a native English speaker; (3) be 10–18 years of age; (4) have normal vision and hearing with correction; (5) be able to use a computer; (6) score <80% correct on an eye gaze screening task; (7) have a full scale IQ of 70–130, assessed using the KBIT‐2 (Kaufman & Kaufman, 2004); and (8) have at least a second grade reading level, assessed by the OWLS‐2 (Carrow‐Woolfolk, 2011). Participants were excluded if (1) they had seizures within previous 2 years; (2) they lacked stable home Internet; or (3) the parent or adolescent refused to or was unable to consent/assent.

#### Randomization

After consent and baseline assessments, participants were randomly assigned to one of the two conditions (serious game treatment vs. standard care control) over a period of 10 weeks by the Principal Investigator (KSS) who did not have any involvement with clinical or experimental procedures. Randomization proceeded according to a computer‐generated list in a 1:1 ratio (serious game treatment, standard care), stratified by gender and full‐scale IQ (>100, <100). All data were stored within electronic databases on secure servers with password‐controlled access.

#### Blinding procedures

Researchers involved in the clinical assessment and data collection procedures were blinded from condition assignment during the pre‐intervention data collection session. Parents and adolescent participants were not blinded from knowing the condition assignment. Researchers involved in ensuring the fidelity of the intervention were not involved in the data collection procedures. Researchers involved in the data collection at the post‐intervention visit were blinded from condition assignment as well. However, in some cases unblinding occurred when participants volunteered information about their experience in the intervention during this visit. Importantly, the primary outcome measures are believed to be robust to investigator bias.

### Measures and assessments

#### SAGA: A serious game intervention

SAGA is an adventure game with embedded serious game techniques in which participants solve puzzles in a 3D environment that is programmed in Unity (https://unity3d.com/unity). The core training mechanisms are delivered via character interactions. Participants learn skills through simulated social interactions with human avatars in the game as they solve problems related to the game narrative. Each social scene has variable elements, including different objects, locations, and levels of difficulty, that are dynamically altered. Moreover, scenes are presented with a variety of characters and environmental contexts to enhance engagement and support generalized learning opportunities. Comprehensive details about the game design and learning mechanisms are explained elsewhere (see Supporting Information  S1).

The game is designed to train learning about three functional uses of eye gaze cues, including the use of gaze to reference locations and objects in the world via a single informant and in episodes of joint attention between multiple informants. Three sequential phases are implemented in the game. Tasks in phase 1 are structured to help participants learn that eye gaze is an important cue to solving problems in the game. Tasks in phase 2 help participants learn to estimate precise gaze trajectories by making potential gazed‐at objects closer together and to ignore salient objects that are not the locus of the gaze cue. Episodes of joint attention are also introduced in phase 2 as participants have to determine the target object that two avatars are looking at together. This is difficult because the timing of the nonverbal cues to identify the object is not perfectly synchronous between the two avatars. In phase 3, tasks are structured around helping participants learn the difference between a goal‐directed gaze cue (e.g., looking at a target object to solve a puzzle) and a non‐goal‐directed gaze cue (e.g., looking around at all the objects before deciding which one to select).

To complete a phase of the game, participants must finish all levels within a phase. Each phase has multiple levels. Levels are defined by the number of non‐verbal cues (e.g., hand pointing, shoulder and head orientation, eye gaze cues) avatars use to guide participants to solve puzzles in the game. All levels include eye gaze cues. Easy levels have additional non‐verbal cues and the progression to subsequent levels increasingly focuses learning to use eye gaze cues exclusively by stripping away the other cues. To complete a level within a phase of the game, participants must finish all states within a level (i.e., stages nested in levels nested in phases). Each stage (*n* = 6) represents the number of potential objects or locations that the participant has to discriminate between based on the gaze cue from the avatar. In the easiest stage (i.e., stage 1), the participant chooses between two objects or locations that the avatar is pointing, directing shoulders, head, and gaze to, whereas in stage 6, the participant chooses between six possible objects or locations that the avatar could be referring to with the non‐verbal cue(s). Within each stage, participants must perform with 80% accuracy to advance to the next stage, and they must finish all stages within a level before they can progress to the next level within a phase. When they perform <80% accuracy within a stage, they return to the previous stage to reify learning where they were recently successful (including, if necessary, returning to later stages of previous levels).

The treatment group was instructed to download the SAGA game on their own computer and play it at home for three 30‐min sessions per week for 10 weeks. Parents were specifically instructed not to let anyone but the participant play the game and to avoid helping the participant unless it was to resolve a technical issue (e.g., restarting the computer or Internet connection). To improve adherence, families were sent reminders via email or text about their schedule of gameplay. To minimize the potential intrusiveness of gameplay on participants’ schooling and family activities, it was programmed to close after 90 min of play on a single day. This “dose” of treatment was estimated based on the tolerance and relative amount of training required to evince learning in prior face‐processing intervention studies in ASD (Damiano et al., 2011). The goal was for participants to obtain at least 10 h of training specifically on eye gaze tasks across the 10‐week training period, which may have required a total of 15–20 h of total gameplay.

#### Autism community involvement

The decision to employ serious game mechanics in the intervention was informed by positive feedback from adolescents on the autism spectrum tested in previous home‐based computerized interventions (Scherf, Whyte, Minshew & Behrmann, “Adolescents with autism learn to individuate novel objects holistically: Replicated Longitudinal Intervention Studies”). The staff training and testing procedures used in this study, including accommodations in the testing rooms (e.g., lighting, seating) and strategies for working with participants, are all informed by experiences and conversations with previous study participants on the autism spectrum. Several adolescents on the autism spectrum provided feedback to us about the intervention game during its development in pilot testing. Participating families have been informed about findings from the study in the form of a newsletter. We thank all the families who helped inform the development of this study.

### Outcomes

#### Feasibility outcomes

We report participant attrition, number of sessions played, total number of hours played, and hours engaged in eye gaze tasks (see Table 1). We also documented any adverse or unexpected events that occurred during the course of the intervention.

**TABLE 1:** Sample characteristics and feasibility outcomes

	Control	ITT	AT
*n*	20	20	14
Age, mean (SD), mo	163.8 (31.9)	165.8 (33.7)	168.14 (36.9)
Sex, No. (%)			
Male	17 (85.0)	16 (80.0)	12 (85.7)
IQ, mean (SD)			
FSIQ	99.7 (13.4)	100.5 (16.6)	103.3 (18.3)
VIQ	98.0 (18.5)	97.0 (15.4)	99.0 (17.0)
PIQ	100.6 (10.8)	104.0 (16.0)	107.0 (17.1)
ADOS‐2, mean (SD)			
Total	13.8 (4.2)	13.4 (5.0)	13.4 (5.6)
Social affect	10.6 (3.9)	9.6 (3.9)	9.7 (4.5)
Restricted and repetitive behavior	3.3 (1.7)	3.8 (1.6)	3.6 (1.5)
Feasibility outcomes			
Retention rate, No. (%)	20 (100)	20 (91)	14 (100)
Adverse events, No. (%)	0 (0)	0 (0)	0 (0)
Total gameplay, mean (SD), h	–	17.8 (4.8)	19.1 (4.1)
Total time in gaze tasks, mean (SD), h	–	11.4 (3.9)	13.3 (2.8)

#### Safety outcomes

A Data Safety Monitoring Board (DSMB) was composed of independent researchers who have expertise complementary to the aims of SAGA. We met with the DSMB prior to enrolling participants in the study and biannually during the duration of the intervention to review the safety and tolerance of the intervention for our participants. Any adverse events were reported to both the DSMB and the Penn State Institutional Review Board.

Adverse events and unintended effects during testing were monitored by the research staff. Additionally, self‐report and behavioral measures were used to monitor unanticipated risks. This included a Usability questionnaire about the intervention game experience in which participants rated multiple aspects of gameplay on a Likert scale (e.g., Experience was fun; I felt discouraged) at the post‐intervention testing session. As required by the National Institutes of Health, procedures were in place to monitor suicidal ideation and self‐injurious behavior among adolescents and to make recommendations about care based on the assessment outcome.

#### Primary outcome

To evaluate participants’ ability to process and interpret eye gaze cues, we developed a Gaze Perception task (Bill et al., 2020). Participants viewed images (4000 ms) of actors in naturalistic scenes looking at one of many possible objects. Participants were instructed to identify the object that the person is looking at from a list of four labels presented on a subsequent screen (i.e., multiple choice). Success in this task requires that participants can perceive the trajectory of gaze and understand the referential intent of the actor to look at a specific object. The images in the pre‐ and post‐intervention testing sessions included 65% novel images and 35% repeated images. Importantly, of these repeated images, the percentage of agreement at pre‐ and post‐intervention in the standard care control group was 72%, indicating moderate test–retest–reliability (Svensson, 2012).The primary dependent variable was performance accuracy. Measures of social visual attention to the images were also collected and will be presented in a subsequent manuscript.

#### Secondary outcomes

To assess potential changes in social communication skills and autism social deficits, parents and adolescents each completed the Social Skills Improvement System (SSIS; Greshman & Elliott, 2008) and Social Responsiveness Scale, second edition (SRS‐2; Constantino & Gruber, 2012) at the pre‐ and post‐intervention sessions. The SSIS has been identified as one of the six measures that are appropriate for measuring changes in social communication in people on the autism spectrum, specifically as a treatment endpoint because it is clinically relevant, has reliable and valid psychometric properties for social communication, is sensitive to change, has been used in people on the autism spectrum previously, and has a reasonable level of burden (see Anagnostou et al., 2015). The SSIS provides standard scores with a mean of 100 and an SD of 15 for two domains, Social Skills and Problem Behaviors. Importantly, the SSIS is sensitive to change in studies of behavioral treatment in typically developing children and those with developmental disorders (for review, see Anagnostou et al., 2015).

The SRS‐2 total score (*T*‐score) is a reliable measure for social deficits related to ASD (Constantino & Gruber, 2012). *T*‐scores greater than or equal to 76 indicate clinically significant deficits in social functioning that interfere with social interactions; scores between 66 and 75 indicate some clinically significant social deficits; and scores between 60 and 65 indicate mild to moderate deficiencies in social behavior (Constantino & Gruber, 2012).

### Statistical analysis

Power calculations indicated that with a sample size 17 per group, and an expected correlation between the pretest/posttest measures of 0.58, we would have statistical power of 0.80 to detect a medium effect size for the expected group (serious game treatment, standard care control) × time (pre‐intervention and post‐intervention) interaction when *α* = 0.05 (Riby et al., 2013; Tang et al., 2019).

All analyses were conducted using R (R Core Team, 2020). For accuracy, we used the *lme4* package to fit generalized linear mixed effects models to evaluate treatment effects (group × time interaction; Bates et al., 2015). The model included the fixed factors of Group (treatment, control; reference = control) and Time (pre‐, post‐intervention; reference = pre‐intervention) and the random factors of stimulus item and participant with a random intercept. We used a binomial link function and the Wald method for calculating confidence intervals. All parameter estimates (log odds) were converted to odds ratio for effect size estimation.

To evaluate associations between potential improvements in eye gaze sensitivity and social skills, we computed change scores (i.e., post minus pre‐intervention) in both the eye gaze perception (i.e., accuracy) and parent‐reported measures (i.e., SSIS/SRS‐2) because individuals on the autism spectrum reportedly have limited insight into their own social skills and symptoms (Hill et al., 2004). Difference scores were submitted to linear regression to evaluate whether associations between them differed as a function of group (i.e., differences in the slopes of the regression lines for the two groups).

The main analyses followed an unbiased *intent‐to‐treat* (ITT) principle and included all participants who were randomized and started the intervention (if assigned to the treatment group). This RCT was not designed or powered to evaluate a dose‐dependent response within the treatment group. However, based on the existing literature, we developed an operational definition for a *sufficient dose* of the treatment that motivated a secondary analysis to examine the effectiveness of the intervention in individuals who received this sufficient dose. The *as‐treated* (AT) analysis included participants randomized into the treatment group who completed at least 10 h of training in the eye gaze tasks versus all the participants randomized into the standard care control group (Ellenberg, 1996).

## RESULTS

**FIGURE 1:**
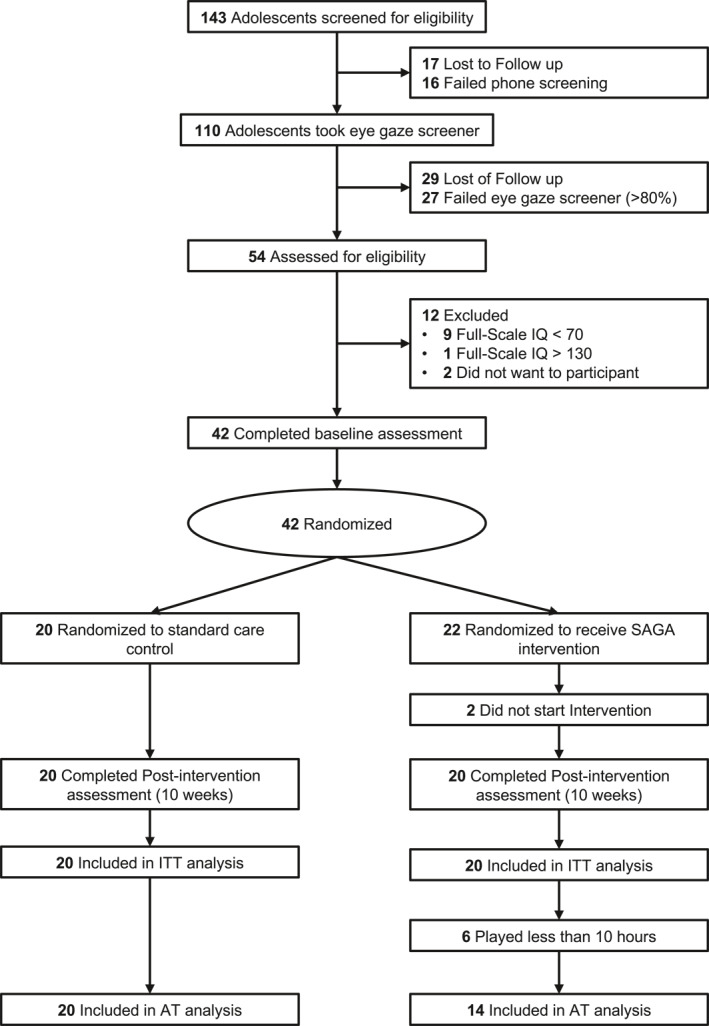
SAGA CONSORT flow diagram. AT, as treated; ITT, intent to treat

Figure 1 shows the CONSORT diagram of the SAGA protocol. A total of 143 families were assessed remotely for eligibility, 110 of whom were eligible and invited to complete the online eye gaze screening task. Of this group, 82 of 110 participants who completed the screening task exhibited deficient eye gaze processing (i.e., a score greater than half SD below the mean of a group of typically developing adolescents on same task; Scherf et al., 2018). A total of 54 of these families traveled to PSU for a full eligibility assessment. 12 participants were excluded prior to pre‐intervention assessments. A total of 42 adolescents on the autism spectrum were enrolled and successfully randomized into the study. Two families were eligible for the study, but after completing the pre‐intervention assessments, were not interested in starting the intervention or participating in the remainder of the study. Therefore, these participants were replaced after randomization. As a result, a total of 40 participants completed all aspects of the study. The ITT treatment sample included 20 participants and the AT intervention sample included 14 participants.

### Feasibility

Table 1 shows adherence to and engagement with the intervention. Participants were instructed to play SAGA a minimum of 15 h over the 10‐week period. The ITT sample exceeded this request (*M* = 17.8 total hours, SD = 4.8), as did the AT sample (*M* = 19.1 total hours, SD = 4.1). Within the game, engagement in eye gaze tasks was greater than 10 h (ITT: *M* = 11.4 h, SD = 3.9; AT: *M* = 13.3 h, SD = 2.8).

### Safety

There were no adverse or unexpected events. Using a Likert scale in which 1 = strongly disagree, 3 = neutral, and 5 = strongly agree, intervention participants indicated largely neutral responses to the usability questions following the intervention experience: including, “Experience was fun” (*M* = 2.9, SD = 1.3), “Experience was rewarding” (*M* = 3.3, SD = 1.3), “I felt discouraged” (*M* = 2.7, SD = 1.3), and “I was frustrated” (*M* = 3.0, SD = 1.5).

### Primary outcome measure—Gaze perception task accuracy

#### Intent to treat

**FIGURE 2:**
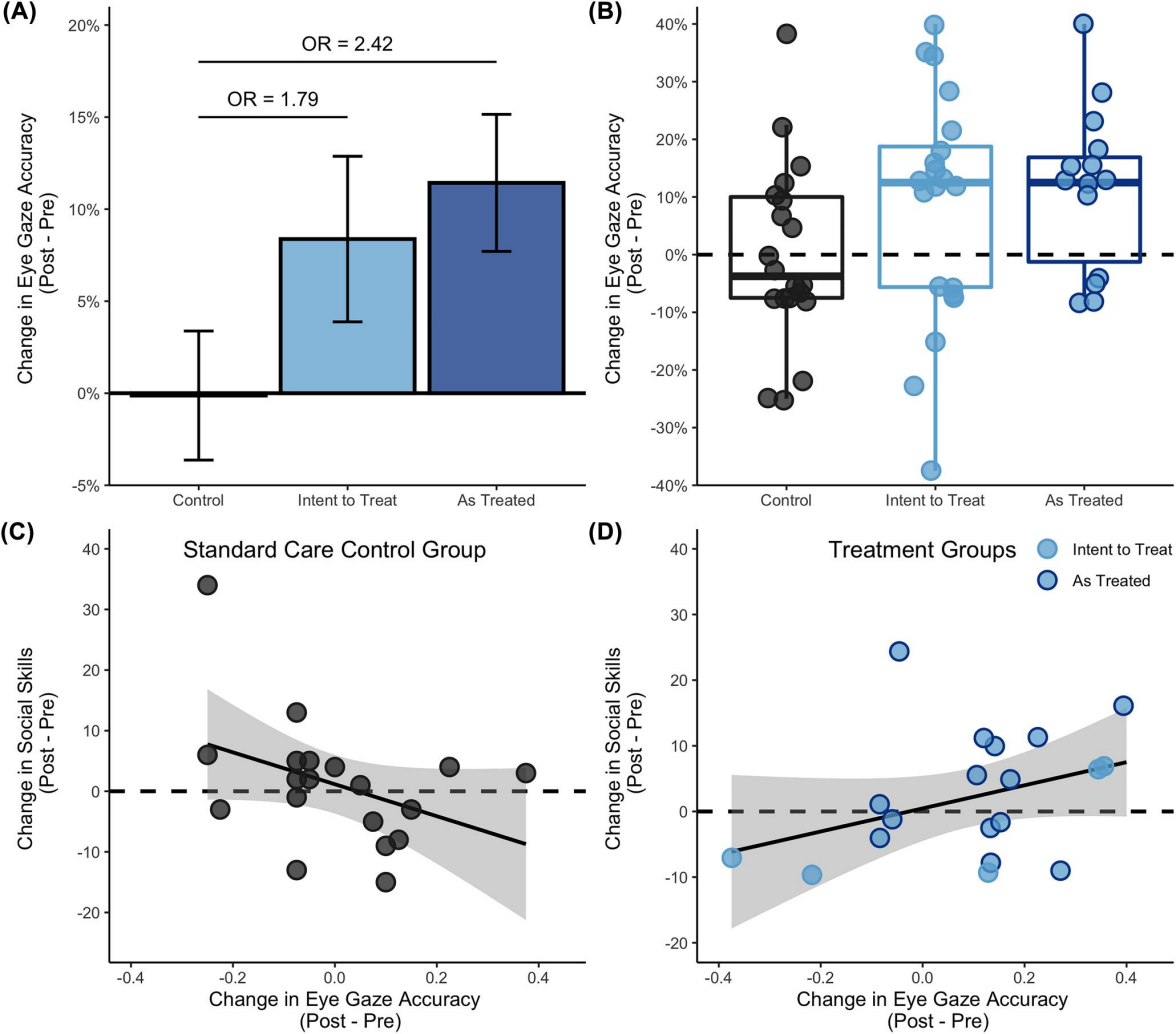
Change in primary and secondary outcomes following SAGA. (A and B) Accuracy in the Eye Gaze Perception task plotted as a change (i.e., difference) score over time (±1 SEM). As a group, adolescents in the intent‐to‐treat group (light blue bar) improved their ability to detect and interpret eye gaze cues (primary outcome) by ∼7%. Adolescents in the as‐treated group (dark blue bar), who engaged in eye gaze tasks for at least 10 h during the intervention, improved in the primary outcome by ∼12%. (B) Box and whisker (minimum, interquartile range, maximum) plot showing the range of individual participant change scores in primary outcome as a function of group. (C and D) Change scores in the Eye Gaze Perception task plotted as a function of change in parent‐reported social skills for the treatment (C) and standard care control (D) groups (with 95% CI). Larger improvements in sensitivity to the eye gaze cues were related to larger positive changes in parent‐reported social skills, but only for the intent‐to‐treat group (D). A small amount of random variation (i.e., jitter) was added to individual points to minimize overplotting

Figure 2a and 2b show the change in performance over time as a function of group and separately for each individual within each group. There was a significant treatment effect (group × time interaction: OR = 1.79, 95% CI [1.22, 2.64], *p* = .003; see Tables 2 and 3). Specifically, the treatment group showed large improvement across time (OR = 1.77, 95% CI [1.21, 2.57], *p* = .003), whereas the standard care control group did not (OR = 0.84, 95% CI [0.57, 1.23], *p* = .36). We also explored an additional analysis including random slopes, which resulted in an even larger treatment effect (OR = 1.91), but increased variability around the average effect (see Table S1).

**TABLE 2:** Primary and secondary outcomes over time

	Pre‐intervention			Post‐intervention		
	Control (*n* = 20)	ITT (*n* = 22)	At (*n* = 14)	Control (*n* = 20)	ITT (*n* = 20)	At (*n* = 14)
Primary outcome						
Task performance	81.0 (10.4)	76.5 (12.7)	76.8 (10.8)	80.9 (10.4)	84.9 (12.4)	88.2 (6.6)
Secondary outcomes						
SSIS—social skills	84.1 (13.1)	75.0 (14.2)	75.8 (12.6)	85.2 (10.9)	77.4 (15)	79.8 (10.1)
SSIS—problem behaviors	118.1 (11.9)	120.6 (15.1)	121.4 (13.1)	118.0 (11.5)	124.2 (14.2)	126.6 (12.7)
SRS‐2	74.1 (9.8)	77.0 (9.3)	76.0 (8.0)	73.25 (9.1)	74.5 (8.8)	73.9 (7.2)

*Note*: Cells contain group mean (SD).

Abbreviations: AT, as‐treated sample (received at least 10 h of training in eye gaze tasks); ITT, intent‐to‐treat sample; SRS‐2, Social Responsiveness Scale (second edition); SSIS, Social Skills Improvement System.

**TABLE 3:** Results from analyses of treatment effects

Intent to treat		
Primary outcome, OR [95% CI]	Estimate	*p*
Task performance		
Treatment effect (group × time)	1.79 [1.22, 2.64]	.003
Standard care control	0.84 [0.57, 1.23]	.36
Treatment	1.77 [1.21, 2.57]	.003

Secondary outcomes, *b* [95% CI]		
SSIS—social skills		
Treatment effect (group × change score)	43.90 [7.18, 80.60]	.02
Standard care control	−26.30 [−57.20, 4.53]	.09
Treatment	17.60 [−5.38, 40.50]	.13
SSIS—problem behaviors		
Treatment effect (group × change score)	10.8 [−27.10, 48.70]	.57
Standard care control	−4.29 [−37.80, 29.30]	.79
Treatment	6.53 [−15.40, 28.50]	.54
SRS‐2		
Treatment effect (group × change score)	−8.24 [−32.20, 15.70]	.49
Standard care control	11.80 [−11.50, 35.10]	.30
Treatment	3.58 [−8.36, 15.50]	.54
As treated		

Primary outcome, OR [95% CI]	Estimate	*p*
Task performance		
Treatment effect (group × time)	2.42 [1.55, 3.78]	<.001
Standard care control	0.84 [0.57, 1.23]	.36
Treatment	2.40 [1.49, 3.87]	<.001

Secondary outcomes, *b* [95% CI]		
SSIS—social skills		
Treatment effect (group × change score)	34.6 [−15.80, 84.90]	.17
Standard care control	−26.30 [−57.20, 4.53]	.09
Treatment	8.23 [−34.90, 51.30]	.69
SSIS—problem behaviors		
Treatment effect (group × change score)	0.61 [−53.00, 54.20]	.98
Standard care control	−4.29 [−37.80, 29.30]	.79
Treatment	−3.68 [−47.70, 40.40]	.86
SRS‐2		
Treatment effect (group × change score)	0.38 [−33.10, 33.90]	.98
Standard care control	11.80 [−11.50, 35.10]	.30
Treatment	12.20 [−10.20, 34.60]	.26

Abbreviations: AT, As Treated sample (received at least 10 h of training in eye gaze tasks); ITT, Intent to Treat sample; SRS‐2, Social Responsiveness Scale (second Ed); SSIS, Social Skills Improvement System.

Finally, Figure 2b indicates that there is heterogeneity in the treatment response (e.g., some scores increase, and others decrease following the intervention). Of particular concern is the participants who evinced a decrease in performance following the intervention and whether this decrement in performance indicates a harmful effect of the intervention. Alternatively, a decrease in scores over time could reflect a minimal treatment effect in response to an underlying developmental decrement in the core skill. Understanding the relative heterogeneity in the standard care control versus treatment groups will help delineate between these two interpretations.

We conducted non‐parametric sign tests (i.e., Wilcoxon) to evaluate the probability of participant scores changing over time in both groups. Participants in the standard care control group were equally likely to exhibit increasing (*N* = 10) or decreasing (*N* = 11) performance (i.e., sensitivity to eye gaze cues) over time (*V* = 93.5, *p* = .97). However, participants in the treatment group were much more likely to evince an increase (*N* = 13), relative to a decrease (*N* = 7), in performance over time (*V* = 53.5, *p* = .05).

#### As treated

The AT treatment group revealed a greater treatment effect (OR = 2.4, 95% CI [1.49, 3.87], *p* < .001; see Figure 2a). The AT group was also much more likely to exhibit an increase (*N* = 10) than a decrease (*N* = 4) in performance following the intervention (*V* = 10.0, *p* = .008).

### Secondary outcomes–social communication skills, problematic behaviors, social deficits

#### Intent to treat

Prior to the intervention, parents reported that adolescents in both groups (treatment, standard care control) exhibited difficulties with social communication skills and problem behaviors (>1 SD from standard score; see Tables 2 and S2). Parents also reported that adolescents in both groups exhibited clinically significant (i.e., moderate to severe) social deficits. Prior to evaluating the effects of the intervention on these social skills, we evaluated whether there was a differential change in social skills, problem behaviors, or autism social symptoms as a function of group over time. This is especially important to determine since parents were not blind to the experimental condition of their child, which may have influenced their reporting at the post‐intervention time point. Neither group showed a significant change in parent‐reported social skills over time (i.e., no main effect of time or group × time interaction; see Table 3). These findings indicate no differential response bias for parents whose child was assigned to the treatment group.

Second, we evaluated whether individual differences in the magnitude of the treatment effect (i.e., change in task performance over time) were associated with a change in social skills, problem behaviors, or autism symptoms and whether this association varied as a function of group (see Table S2). The regression revealed a significant group × change score interaction (*b* = 43.91, 95% CI [7.2, 80.6], *p* = .02).^1^ Analyses of the simple main effects revealed that improvements in task performance were associated with improvements in social communication skills (as measured by the SSIS), but only in the treatment group (*b* = 17.6, 95% CI [−5.38, 40.5], *p* = .13; see Table 3; Figure 2c and 2d). In contrast, there was no such treatment effect associated with a change in problem behaviors or autism symptoms (see Table 3).

#### As treated

This treatment effect was not replicated in the smaller AT analysis (see Table 3).

## DISCUSSION

SAGA was a feasible, tolerable, safe, and effective intervention for improving sensitivity to eye gaze cues in adolescents on the autism spectrum. There were no adverse or unexpected events. The treatment group was compliant with the home‐based intervention training schedule. There was no participant attrition related to the experience of participating in the intervention, which may be related to the home‐based nature of the intervention and/or our retention strategies. This is notable given that the average attrition rate of computer‐based interventions for ASD is 19.17% (Tang et al., 2019).

Importantly, the treatment group, but not the standard care control group, exhibited improved sensitivity to eye gaze cues in tasks of behavioral generalization. Although participants were trained by computerized avatars to use eye gaze cues in the game, they exhibited improved processing of *human* eye gaze cues in the tasks of generalized learning. Moreover, participants in the treatment group who engaged the eye gaze tasks for at least 10 h during the intervention showed the greatest improvements in eye gaze sensitivity. These findings suggest that the serious game principles of SAGA support learning about the functional significance of eye gaze cues and that adolescents who received a sufficient dose of the intervention showed a larger improvement in the primary outcome.

In addition to improving a core phenotype of ASD, namely a disability in understanding the functional significance of eye gaze cues, this RCT revealed that these improvements were associated with increased social communication skills as reported by parents. Importantly, these results are unlikely to reflect reporting biases from parents in the treatment group because there were no group differences in parent‐reported social skills prior to or following the intervention. In other words, the parents of the intervention participants did not have a systematic bias to report improved social communication skills following the intervention experience compared to the parents of the control participants. Also, although parent reports overwhelmingly indicated that adolescents continued to exhibit deficits in social communication skills (SSIS scores >1 SD beyond standardized mean) following the intervention, some participants did show improvements on the order of one full standard deviation and/or to an extent that reduced the clinical significance of their impairment.

The magnitude of these treatment effects is on par with those identified from meta‐analyses of computer‐based interventions for autism (Grynszpan et al., 2014; Tang et al., 2019). For our primary outcome measure, the effect size of the treatment effect was moderate‐large for the ITT (OR = 1.79) and AT (OR = 2.42) groups. It was a small‐moderate effect size for the secondary outcome measure (ITT Cohen’s *d* = 0.16; AT Cohen’s *d* = 0.38). These are notable effects given the small size of this RCT and provide confidence in this initial test of this intervention approach.

It is important to note that previous studies often employ tests of learning that employ the exact task structure and stimuli used in training. This approach limits understanding of generalization of learning outside the training context. In the current study, participants were trained to implicitly learn about eye gaze cues in a game with computerized avatars. However, the test of effectiveness of the intervention involved assessing referential understanding of *human* eye gaze cues using novel stimuli. This approach provides confidence that learning has transferred outside the context of the game.

SAGA builds upon the evidence that targeting social looking behaviors for intervention in autism has clinical relevance and can alter core symptoms of autism. Interventions that target social looking behaviors, like joint attention and social referencing, have been successful with children and toddlers (see Murza et al., 2016). Our findings support the evidence that without intervention, eye gaze processing remains a significant deficit in autism through adolescence (Griffin & Scherf, 2020). In fact, participants in the standard care control group were equally likely to exhibit a decrease in sensitivity to eye gaze cues over time, indicating that part of the mechanism of action of the intervention may be attenuating a developmental decline in this core social communicative ability in ASD. In contrast, participants in the serious game treatment group were more likely to exhibit an increase in sensitivity to eye gaze cues, particularly if they acquired at least 10 h of training on the eye gaze tasks. Critically, this improvement was associated with progress in their general social communication skills. Therefore, SAGA offers an effective treatment tool to support individuals on the autism spectrum throughout adolescence that is compatible with the social looking behavior interventions that target earlier developmental periods.

## LIMITATIONS

This initial evaluation of the effectiveness of SAGA is embedded in a larger program of research that aims to evaluate the clinical relevance and generalization of learning in real world social interactions. Importantly, the goal of this initial study was to evaluate the feasibility of SAGA as an intervention designed to engage a prespecified target mechanism (the ability to perceive and interpret shifts in eye gaze as social signals) and improve it. Pending success with this initial goal, it will be important to test SAGA against an appropriately matched control game, in a larger sample, to provide converging and more rigorous evidence of its effectiveness. In so doing, it will be important to employ a double‐blind RCT comparing SAGA to a control game. Also, it will be important to evaluate how well untrained behaviors, dynamic social‐communicative interactions, and clinical symptoms change following SAGA. Finally, future work is needed to evaluate the persistence of these improvements.

## CONCLUSIONS

SAGA showed initial promise as an effective tool for improving sensitivity to eye gaze cues and social communication skills in adolescents on the autism spectrum. Although future research is needed to better establish effectiveness and boundary conditions, SAGA has the potential to be an accessible, scalable, and affordable intervention tool that can be used in conjunction with current standard treatments for autism.

## CONFLICT OF INTEREST

The authors have declared that they have no competing or potential conflicts of interest.

## ETHICS STATEMENT

Consent was obtained from all participants and their parent and/or guardian prior to participating. Ethical approval was granted by Pennsylvania State University Institutional Review Board (IRB # 00005097).

## AUTHOR CONTRIBUTIONS

K. Suzanne Scherf, Joshua M. Smyth, and Charles F. Geier conceptualized the study and methodology and acquired funding to support it. K. Suzanne Scherf provided supervision, project administration, and resources for the project. Jason W. Griffin supported the data curation, formal analyses, and visualization of the results. Jason W. Griffin drafted the original manuscript. All authors provided critical review and revisions prior to approving the final version.

## Supporting information

Supporting Information S1

Supporting Information S2

## Data Availability

The data that support the findings of this study will be uploaded to the National Institutes of Health National Data Archive (NDA) https://nda.nih.gov/.

## ENDNOTE

^1^
We also used a linear mixed‐model to address the repeated‐measures nature of this design and found a similar treatment effect.
