# Supplementary material for: Improving sensitivity to eye gaze cues in adolescents on the autism spectrum using serious game technology: A randomized controlled trial
Source: JCPP Adv. 2021 Oct 4;1(3):e12041. doi: 10.1002/jcv2.12041 (PMC9835110; doi:10.1002/jcv2.12041)
Supplement: Supplementary file 1 — Supporting Information S1 [file JCV2-1-e12041-s002.docx]

| **Table S1.** Primary Outcome results with varying random slope of time. | | | | |
| --- | --- | --- | --- | --- |
| **Intent to Treat** | | | | |
| **Primary Outcome, OR [95% CI]** | | | Estimate | *p* |
| *Task Performance* | | |  |  |
|  | Treatment Effect (Group x Time) | | 1.91 [0.82, 3.61] | .13 |
|  | | Standard Care Control | 0.84 [0.46, 1.53] | .58 |
|  | | Treatment | 1.99 [0.95, 4.12] | .065 |
| **As Treated** | | | | |
| **Primary Outcome, OR [95% CI]** | | | Estimate | *p* |
| *Task Performance* | | |  |  |
|  | Treatment Effect (Group x Time) | | 2.40 [1.49, 3.87] | **<0.001** |
|  | | Standard Care Control | 0.84 [0.46, 1.53] | 0.58 |
|  | | Treatment | 2.42 [1.22, 4.79] | **0.01** |
| *Note.* ITT = Intent to Treat sample; AT = As Treated sample (received at least 10 hours of training in eye gaze tasks); SSIS = Social Skills Improvement System; SRS-2 = Social Responsiveness Scale (2^nd^ Ed) | | | | |

# **Supporting Information**

| **Table S2.** | | | | | | | | | | | | | | | |
| --- | --- | --- | --- | --- | --- | --- | --- | --- | --- | --- | --- | --- | --- | --- | --- |
| *Secondary Outcome Measures for Treatment and Standard Control Participants, including the SSIS-Social Skills, SSIS-Problem Behaviors, and SRS-2 Total Scores.* | | | | | | | | | | | | | | | |
| **Treatment** | | | | | | | | **Standard Care Control** | | | | | | | |
| ID | ΔEye Gaze | SSIS  Social Skills | | SSIS  Problem Behaviors | | SRS-2 | | ID | ΔEye Gaze | SSIS  Social Skills | | SSIS  Problem Behaviors | | SRS-2 | |
|  |  | Pre | Post | Pre | Post | Pre | Post |  |  | Pre | Post | Pre | Post | Pre | Post |
| 1 | -0.37 | 51 | 43 | **117** | **108*** | 87 | 83 | 1 | -0.25 | **53** | **87*** | 107 | 114 | **80** | **63^c^** |
| 2 | -0.22 | 64 | 55 | 136 | 138 | 87 | 88 | 2 | -0.25 | 89 | 95 | **119** | **110*** | 62 | 74^b^ |
| 3 | -0.15 | 70 | NA | 118 | 119 | **82** | **74^b^** | 3 | -0.22 | 86 | 83* | 119 | 129 | 75 | 73 |
| 4 | -0.07 | 67 | 69 | 102 | 108 | 73 | 73 | 4 | -0.08 | 79 | 81 | 111 | 117* | 74 | 70 |
| 5 | -0.07 | 76 | 71 | 125 | 144 | 82 | 78 | 5 | -0.08 | 89 | 102 | **120** | **109*** | 68 | 67 |
| 6 | -0.05 | 43 | 67 | 143 | 146 | 90 | 80 | 6 | -0.08 | 91 | 96 | 121 | 118 | 74 | 71 |
| 7 | -0.05 | 84 | 82 | 121 | 121 | **80** | **75^b^** | 7 | -0.07 | 72 | 71 | 136 | 139 | 87 | 77 |
| 8 | 0.10 | 80 | 85 | 107 | 128* | 66 | 75 | 8 | -0.07 | 93 | 80* | 103 | 115* | 72 | 83^a^ |
| 9 | 0.12 | 85 | 77 | 140 | 140 | 72 | 70 | 9 | -0.05 | 62 | 64 | 124 | 136 | 80 | 82 |
| 10 | 0.12 | 69 | 80 | **119** | **108*** | 70 | 66 | 10 | -0.05 | 97 | 102 | 102 | 106 | 58 | 57 |
| 11 | 0.12 | 79 | 77 | 123 | 129 | 83 | 79 | 11 | -0.03 | NA | 85 | 132 | 119 | **89** | **74^b^** |
| 12 | 0.12 | 90 | 80 | 100 | 105 | 72 | 75 | 12 | 0.00 | 72 | 76 | 110 | 104 | 78 | 82 |
| 13 | 0.15 | 95 | 93 | 110 | 108 | 64 | 60 | 13 | 0.05 | 89 | 90 | 149 | 143 | 90 | 90 |
| 14 | 0.15 | 69 | 78 | 130 | 126 | 85 | 83 | 14 | 0.08 | 92 | 87 | 113 | 113 | 61 | 64 |
| 15 | 0.18 | 92 | 97 | 109 | 130* | 70 | 68 | 15 | 0.10 | 80 | 65* | **130** | **102*** | NA | 62 |
| 16 | 0.23 | 73 | 84 | 130 | 130 | **80** | **70^a^** | 16 | 0.10 | 101 | 92 | 110 | 114 | 63 | 69^b^ |
| 17 | 0.28 | 73 | 64 | 134 | 135 | 81 | 87 | 17 | 0.12 | 105 | 97 | **117** | **108*** | 64 | 66^b^ |
| 18 | 0.35 | 63 | 69 | 147 | 141 | 90 | 83 | 18 | 0.15 | 90 | 87 | 103 | 119* | 70 | 72 |
| 19 | 0.35 | 101 | 107 | 94 | 101 | 57 | 53 | 19 | 0.22 | 73 | 77 | 119 | 127 | 85 | 90 |
| 20 | 0.40 | **76** | **93*** | 107 | 119* | 68 | 70 | 20 | 0.38 | **85** | **88*** | 117 | 118 | 78 | 79 |
| *Note.* ΔEye Gaze reflects the change score in task performance on the eye gaze task (Post - Pre). SSIS Post scores marked with an * reflect a 1 SD unit increase/decrease from pre-intervention. Bolded SSIS and SRS-2 scores reflect a categorical *improvement* from pre- to post-intervention.  ^a^SRS-2 Post score in the severe range (>75)  ^b^SRS-2 Post score in the moderate range (75-66)  ^c^SRS-2 Post score in the moderate range (65-60) | | | | | | | | | | | | | | | |
